# Physiological effects of high-flow nasal cannula therapy in preterm infants

**DOI:** 10.1136/archdischild-2018-316773

**Published:** 2019-05-23

**Authors:** Zheyi Liew, Alan C Fenton, Sundeep Harigopal, Saikiran Gopalakaje, Malcolm Brodlie, Christopher J O’Brien

**Affiliations:** 1 Paediatric Respiratory Medicine, Great North Children’s Hospital, Newcastle upon Tyne Hospitals NHS Foundation Trust, Newcastle upon Tyne, UK; 2 Institute of Cellular Medicine, Newcastle University, Newcastle upon Tyne, UK; 3 Newcastle Neonatal Service, Newcastle upon Tyne Hospitals NHS Foundation Trust, Newcastle upon Tyne, UK; 4 Newcastle University, Newcastle upon Tyne, UK

**Keywords:** neonatology, respiratory, high flow nasal cannula oxygen, physiology

## Abstract

**Objective:**

High-flow nasal cannula (HFNC) therapy is increasingly used in preterm infants despite a paucity of physiological studies. We aimed to investigate the effects of HFNC on respiratory physiology.

**Study design:**

A prospective randomised crossover study was performed enrolling clinically stable preterm infants receiving either HFNC or nasal continuous positive airway pressure (nCPAP). Infants in three current weight groups were studied: <1000 g, 1000–1500 g and >1500 g. Infants were randomised to either first receive HFNC flows 8–2 L/min and then nCPAP 6 cm H_2_O or nCPAP first and then HFNC flows 8–2 L/min. Nasopharyngeal end-expiratory airway pressure (pEEP), tidal volume, dead space washout by nasopharyngeal end-expiratory CO_2_ (pEECO_2_), oxygen saturation and vital signs were measured.

**Results:**

A total of 44 preterm infants, birth weights 500–1900 g, were studied. Increasing flows from 2 to 8 L/min significantly increased pEEP (mean 2.3–6.1 cm H_2_O) and reduced pEECO_2_ (mean 2.3%–0.9%). Tidal volume and transcutaneous CO_2_ were unchanged. Significant differences were seen between pEEP generated in open and closed mouth states across all HFNC flows (difference 0.6–2.3 cm H_2_O). Infants weighing <1000 g received higher pEEP at the same HFNC flow than infants weighing >1000 g. Variability of pEEP generated at HFNC flows of 6–8 L/min was greater than nCPAP (2.4–13.5 vs 3.5–9.9 cm H_2_O).

**Conclusions:**

HFNC therapy produces clinically significant pEEP with large variability at higher flow rates. Highest pressures were observed in infants weighing <1000 g. Flow, weight and mouth position are all important determinants of pressures generated. Reductions in pEECO_2_ support HFNC’s role in dead space washout.

**What is already known on this topic?**

- High-flow nasal cannula (HFNC) therapy has been rapidly adopted and is increasingly used in preterm infants.
- Mechanisms of action of HFNC are poorly understood; previous studies have found conflicting results, used varied methodology and have included very few infants weighing <1000 g.
- Reduction of dead space ventilation is thought to be one of the mechanisms of action of HFNC but this has not been demonstrated in preterm infants.

**What this study adds?**

- We prospectively evaluated the physiological effects of a range of HFNC flow rates from 2 to 8 L/min in preterm infants, including a substantial number weighing <1000 g.
- The airway pressure generated during HFNC is dependent on multiple factors, including increasing with flow rate; considerable variability was demonstrated.
- Physiological effects of HFNC include reduction in dead space ventilation, respiratory rate and improved oxygenation.

## Introduction

High-flow nasal cannula (HFNC) therapy is increasingly used in preterm infants; perceived benefits include ease of use, increased comfort and bonding.^1^ Systematic reviews have concluded that HFNC has similar efficacy to other non-invasive respiratory support in preterm infants >28 weeks gestation.^2 3^ However, as primary support in respiratory distress syndrome, two recent randomised controlled trials found HFNC to be inferior to nasal continuous positive airway pressure (nCPAP).^4 5^ There is wide variation in the clinical use of HFNC, for example, flow rates and weaning strategies.^1^ This may be partly explained by a lack of understanding of HFNC’s mechanisms of action in neonates.^6^

The few physiological studies performed have involved differing flow rates and measurement techniques, small sample sizes and some only in vitro models.^7^ These have produced conflicting conclusions about pressures generated, relationships with infant weight, mouth leak and comparisons with nCPAP.^8–15^ Furthermore, the ability of HFNC to wash out airway dead space in infants has been proposed as a major physiological mechanism but not demonstrated in preterm infants.^6 16^ There are minimal data on infants weighing <1000 g despite frequent use of flows of up to 8 L/min with uncertainty about airway pressures generated.^8 9^

In this study, we comprehensively evaluated the physiological effects of a range of HFNC flows including airway pressures, dead space washout, tidal volume, minute ventilation and gas exchange, compared with nCPAP 6 cm H_2_O.

## Methods

### Study design

**Figure 1:**
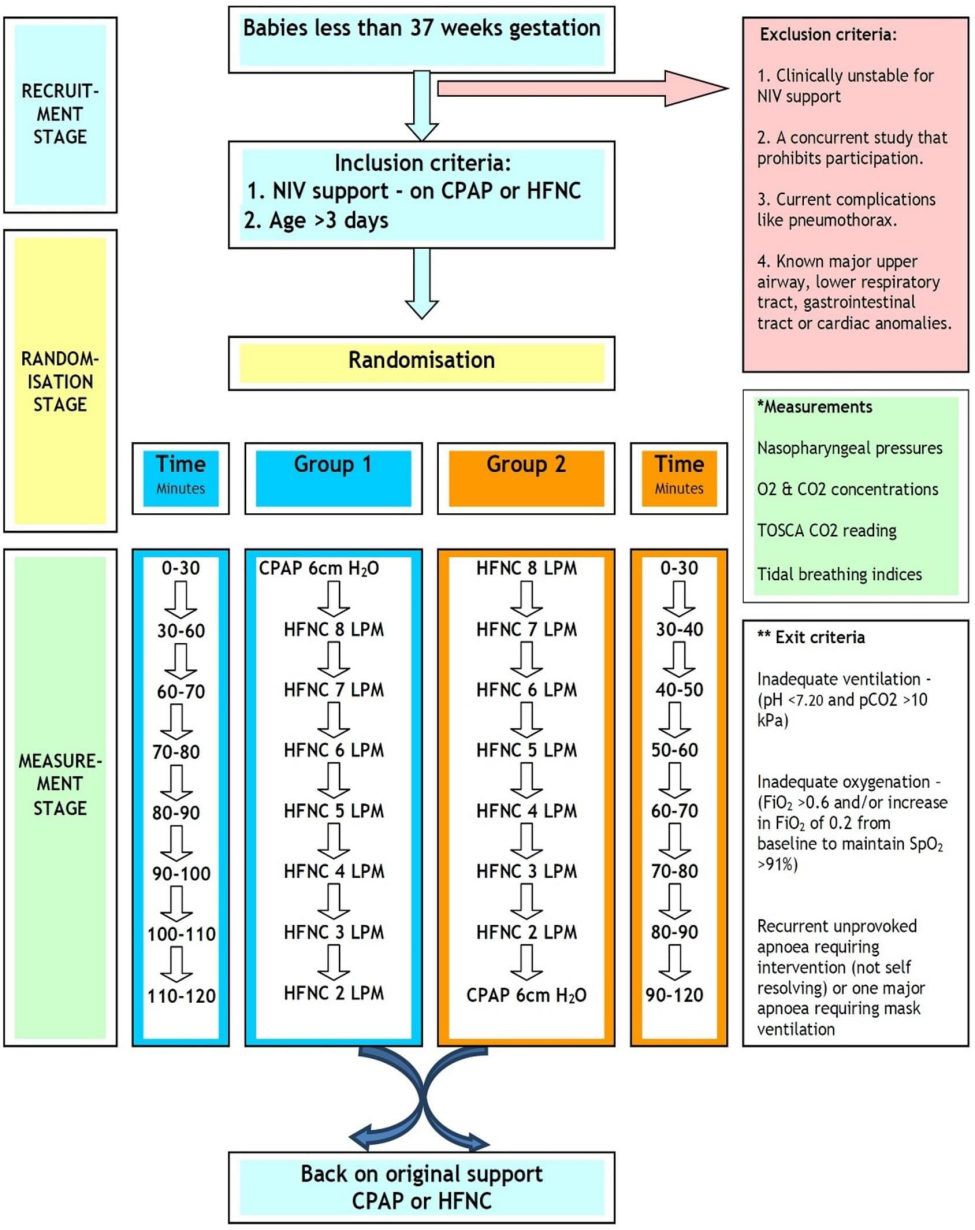
Study flow chart and pathway. Detailed study design and procedures including inclusion, exclusion and exit criteria. CPAP, continuous positive airway pressure; FiO2, oxygen concentration; HFNC, high-flow nasal annula; LPM, litres per minute; NIV, non-invasive ventilation; SpO2, oxygen saturation;TOSCA, transcutaneous CO2.

Prospective randomised crossover study in a tertiary neonatal unit (clinical trials.gov NCT02200900 pre-results). Written informed consent was obtained from parents. A volunteer sample of stable infants <37 weeks gestation, aged >3 days and receiving nCPAP or HFNC for the preceding 12 hours were randomised to group 1 (nCPAP then HFNC) or group 2 (HFNC then nCPAP, see figure 1). The study design was developed with Newcastle and North Tyneside Research Ethics Committee (14/NE/0093) to balance acquisition of the best quality data against the potential for destabilisation in this vulnerable patient group. HFNC flows were adjusted and measurements repeated in a set sequence by 1 L/min to avoid large pressure changes and destabilisation (figure 1). Measurements during nCPAP were performed at a set pressure of 6 cm H_2_O. The timing of studies was arranged to avoid feeds and were delayed ≥30 min during transition between modes and at study entry (see online supplementary methods and figure S1).

10.1136/archdischild-2018-316773.supp1 Supplementary data

### Study size and statistical analysis

Sample size was calculated with airway pressure as the primary outcome using data from previous studies (Minitab V.17).^8 11^ Infants were stratified into current weight groups <1000 g, 1000–1500 g and >1500 g. Twelve infants in each group provided adequate sample size to detect a pressure difference of 0.4 cm H_2_O between flow rates with 80% power and type 1 error of 0.05. An additional three infants per group compensated for study dropouts. See online data supplement for statistical tests used.

### Data sources and measurement

The Fabian Therapy Evolution (Acutronic Medical) provided HFNC and nCPAP. Nasal prongs (NeoFlow, Armstrong Medical) were fitted and inserted as per manufacturer’s recommendation to allow leak around prongs and connected to an AquaVent-Neo breathing circuit (Armstrong Medical) with standard humidification (MR850, Fisher and Paykel). Nasal prongs and diameter of nares were ascertained using a measurement tape. The nCPAP interface used was the IHCA600 (Armstrong Medical) fitted to optimise seal. Humidification was provided during nCPAP using the same humidifier. Nasopharyngeal end-expiratory airway pressure (pEEP) was measured using a suction catheter with two distal side holes (Argyle Gentle Flow 6/8Fr, Covidien) connected to a pressure transducer (B&D Electromedical, range 0–30 cm H_2_O). A 50 mL/hour microinfuser airflow applied at the catheter inlet avoided occlusion. For details of placement see online supplementary figure S2. Dead space washout was evaluated by measuring nasopharyngeal end-expiratory CO_2_ concentration (pEECO_2_) using an analyser (AD Instruments) and the same catheter.

As previously described, mouth position was recorded as ‘open naturally’ or ‘closed’ (pacifier inserted to create a seal, finger lift under chin or naturally closed) at each HFNC flow rate, but not during nCPAP as the primary focus was airway physiology during HFNC therapy.^8^

Tidal volume changes were measured by electromagnetic inductance plethysmography (VoluSense), previously validated in preterm infants (online supplementary methods).^17^

Transcutaneous CO_2_ (TOSCA 500 monitor, Radiometer Medical ApS), oxygen saturation and heart rate (Masimo pulse oximeter) were recorded.

Premeasurement transducer and analyser calibration were performed (online supplementary methods). A multichannel recorder (PowerLab, AD Instruments) allowed synchronised recording and graphical presentation of data, applied sampling frequency 100 Hz (online supplementary figure S3).

### Data extraction and analysis

A 1 min stabilisation period without data extraction followed each respiratory support adjustment. All artefact-free breaths (each selected block containing ≥10 consecutive breaths, online supplementary figure S4) at each step were analysed.

## Results

### Participants

Forty-eight eligible infants were recruited. Data from the first three infants were not analysed due to technical problems with pEEP measurement technique; results from one infant were unanalysable due to missing data. Table 1 details the characteristics of participants; 27 (61%) were male. For baseline respiratory support settings see online supplementary table S1.

**Table 1:** Characteristics of infants in each weight category

Weight category	<1000 g (n=15)		1000–1500 g (n=15)		>1500 g (n=14)		All infants	
	Mean	Median (range)	Mean	Median (range)	Mean	Median (range)	Mean	Median (range)
Birth gestation (weeks)	27.0	27.6 (23.1–30.4)	27.2	27.6 (23.6–31.1)	26.8	26.7 (23.3–31.6)	27.0	26.9 (23.1–31.6)
Current gestation (weeks)	30.4	30.1 (28.3–33.3)	31.7	31.6 (29.9–34.3)	35.6	34.3 (31.1–42.1)	32.5	31.8 (28.3–42.1)
Age (days)	26.9	15 (4–87)	32.9	36 (3–76)	61.6	58 (5–132)	40	35 (3–132)
Birth weight (g)	750	720 (500–1140)	970	920 (500–1440)	970	850 (520–1900)	890	850 (500–1900)
Current weight (g)	880	910 (610–1000)	1310	1250 (1140–1500)	2150	1870 (1520–4200)	1430	1250 (610–4200)

### Generated pEEP at different HFNC flow rates

**Figure 2:**
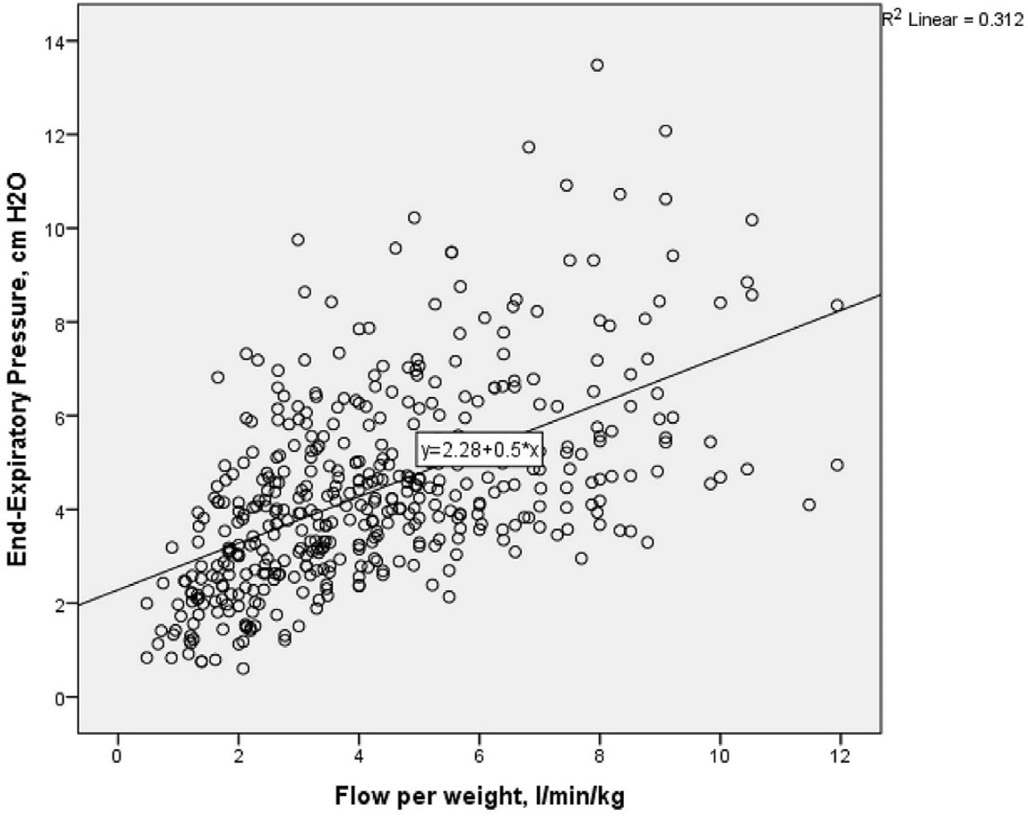
Scatter plot of relationship between nasopharyngeal end-expiratory positive pressure (pEEP) and weight-adjusted flow rate. Figure demonstrates large variability of pEEP measured above 6 L/min/kg, with some pEEP measured up to 8–13 cm H_2_O.

Table 2 shows pEEP generated at each level of support. There was a positive correlation between pEEP and flow rate (r_s_=0.589, p<0.0001). On average, pEEP increased by 0.6 cm H_2_O for each 1 L/min flow rate increment in HFNC (R^2^=0.311, 95% CI 0.47 to 0.61). Figure 2 shows variability in pEEP generated, especially at higher flows. The SD and range of pEEP generated at flows >6 L/min was greater than nCPAP 6 cm H_2_O (range 2.4–13.5 compared with 3.5–9.9 cm H_2_O).

**Table 2:** pEEP at each respiratory support level including effect of mouth position

	HFNC							nCPAP
Flow (L/min)	2	3	4	5	6	7	8	6 cm H_2_O
pEEP (cm H_2_O)	2.3±1.3	3.4±1.6	4.1±1.6	4.2±1.4	4.8±1.7	5.4±2.0	6.1±2.1	6.4±1.5
Mouth closed	2.7	4.0	4.8	5.1	5.7	6.4	7.3	n/a
Mouth open	2.1	2.9	3.3	3.5	4.2	4.5	5.1	n/a
Difference	0.6	1.1	1.5	1.6	1.4	1.9	2.3	n/a
P value*	0.002	0.0001	0.0001	0.0001	0.0001	0.0001	0.0001	n/a
pEECO_2_ (%)	2.3±1.6	1.9±1.5	1.7±1.5	1.7±1.7	1.4±1.5	1.0±1.3	0.9±1.1	2.4±1.8
Vt/kg (mL/kg)	4.3±1.9	3.8±2.0	4.0±1.9	4.4±2.3	3.9±1.6	3.9±1.6	4.2±1.8	4.7±2.1
RR (bpm)†	70±17	64±15	66±18	64±17	63±18	61±16	62±15	66±17
MV (mL/kg/min)	309±162	235±122	258±128	269±157	247±133	239±99	268±148	315±176
TCCO_2_ (kPa)	6.2±1.1	6.2±0.8	6.1±0.9	6.1±1.1	6.1±1.0	6.3±1.0	6.3±0.9	6.5±1.1
S_p_O_2_ (%)‡	92.0±4.4	93.5±3.8	94.2±4.0	94.8±3.5	95.3±3.0	95.9±3.2	96.4±3.3	95.1±3.8
HR (bpm)	156±13	158±12	159±12	160±12	160±10	162±12	164±12	165±13

Effects of HFNC therapy on pEECO_2_, tidal volume, ventilation, gas exchange and haemodynamics.

Expressed as means±SD.

*Wilcoxon signed rank test (mouth position).

†Analysis of variance, p=0.047, when HFNC 8 L/min reduced to HFNC 2 L/min across all flows.

‡Friedman, p≤0.0001, when HFNC 8 L/min reduced to HFNC 2 L/min across all flows.

HR, heart rate; HFNC, high-flow nasal cannula; n/a, not available; nCPAP, nasal continuous positive airway pressure; MV, minute vol; pEEP, nasopharyngeal end-expiratory pressure; pEECO2, nasopharyngeal end-expiratory CO2; RR, respiratory rate; SpO2, oxygen saturation; TCCO2, transcutaneous CO2; Vt, tidal volume.

### Effect of mouth position on HFNC

Generated pEEP was influenced by mouth position, being significantly higher (difference 0.6–2.3 cm H_2_O, p<0.05) with mouth closed, across all flow rates (table 2).

### Effect of weight

Weight was negatively correlated (r_s_=−0.247, p<0.0001) with pEEP; on average decreasing by 0.7 cm H_2_O (95% CI −0.9 to −0.3, p<0.0001) for each kg increase. Table 3 demonstrates the pEEP received by infants in each weight category. Overall, pEEP generated was higher in smaller infants at all flows compared with larger infants (pEEP received in 1000 g group >1000–1500 g>1500 g). Generated pEEP reached 8–13 cm H_2_O at higher flows in some infants (figure 2).

**Table 3:** Comparison of generated nasopharyngeal end-expiratory pressure (pEEP) and nasopharyngeal end-expiratory carbon dioxide concentration (pEECO_2_) in each weight group and flow rate

		pEEP		pEECO_2_	
HFNC flow rate (L/min)	Weight category (g)	Mean±SD	P value*	Mean±SD	P value†
2	<1000	3.0±1.6	0.021	1.6±1.3	0.014
	1000–1500	2.3±1.2		2.2±1.7	
	>1500	1.8±0.7		3.2±1.5	
3	<1000	4.2±1.9	0.005	1.10±0.99	0.003
	1000–1500	3.2±1.5		1.73±1.54	
	>1500	2.6±0.6		2.96±1.41	
4	<1000	5.0±1.9	0.005	0.6±0.6	0.001
	1000–1500	3.6±1.3		2.0±1.6	
	>1500	3.4±0.9		2.5±1.4	
5	<1000	4.6±1.5	NS	0.7±0.7	0.002
	1000–1500	4.0±1.2		2.0±2.1	
	>1500	3.9±1.6		2.5±1.6	
6	<1000	5.5±2.2	NS	0.5±0.8	<0.0001
	1000–1500	4.4±1.2		1.3±1.5	
	>1500	4.5±1.3		2.2±1.5	
7	<1000	5.9±2.5	NS	0.2±0.3	<0.0001
	1000–1500	5.1±1.4		1.1±1.6	
	>1500	5.1±1.8		1.9±1.5	
8	<1000	6.6±2.5	NS	0.2±0.4	<0.0001
	1000–1500	6.0±2.0		1.2±2.1	
	>1500	5.8±1.8		1.8±1.5	

Infants weighing <1000 g n=15, 1000–1500 g n=15, >1500 g n=14.

Expressed in means±SD.

*Jonckheere-Terpstra test for ordered alternatives showed that there was a statistically significant trend of higher pEEP in infants weighing <1000 g compared with infants 1000–1500 g and/or >1500 g at flows 2–4 L/min.

†Jonckheere-Terpstra test for ordered alternatives showed that there was a statistically significant trend of lower pEECO_2_ in infants weighing <1000 g compared with larger weight groups infants 1000–1500 g and/or >1500 g across all flows.

NS, non-significant.

### Effect of prong-to-nares ratio

pEEP and prong-to-nares ratio were positively correlated (r_s_=0.165, p<0.0001). These ratios were further divided into high-leak and low-leak groups (<0.7 and >0.7). Generated pEEP was statistically significantly higher in the low-leak compared with the high-leak group at flows 2–4 L/min (p<0.05, online supplementary figure S5). We consistently observed a drop in pEEP generated if the nasal prongs became partially dislodged during measurements.

### Analysis of factors that affect pEEP generated

On multiple linear regression flow rate, mouth position, current weight and gestation but not prong-to-nares ratio significantly predicted pEEP and account for a significant amount of its variance (F(4431)=143.768, p<0.0001), R^2^=0.572, R^2^=adjusted 0.568). Flow rate was the most significant independent variable, followed by mouth position, weight and current gestation. Predicted pEEP generated=−6.373+0.525×(flow rate, L/min)+1.454×(mouth position, 0=open and 1=closed)−1.856×(weight (kg))+0.307×(current gestation (weeks)).

### Comparison of pEEP generated by HFNC versus nCPAP

Mean pEEP with nCPAP 6 cm H_2_O across all weight groups was 6.4 cm H_2_O (95% CI 6.0 to 6.7); higher than HFNC 2–7 L/min (p<0.05) and comparable to HFNC 8 L/min. However, specifically in infants weighing <1000 g, the mean pEEP with nCPAP 6 cm H_2_O was 5.4 cm H_2_O, similar to that generated by HFNC in the 4–6 L/min range but statistically higher than with HFNC at flows of 2–3 L/min. Importantly, in infants weighing <1000 g pEEP generated by HFNC 7–8 L/min was higher than nCPAP 6 cm H_2_O.

### Dead space washout effect

Despite a clear pressure respiratory waveform, confirmed catheter patency and satisfactory position, pEECO_2_ was often markedly attenuated at higher flows, supporting a significant washout effect. There was a strong, negative correlation between pEECO_2_ and weight-corrected flow rate (r_s_=−0.323, p<0.0001). Open mouth state was associated with greater washout effect (lowered pEECO_2_ measured during mouth open), especially at high flow rates though was not statistically significant (online supplementary table S2). Current weight and pEECO_2_ were positively correlated (r_s_=0.484, p<0.0001). The reduction of pEECO_2_ was greatest in infants weighing <1000 g, and was statistically significant compared with the other 2 weight groups (table 3). The mean nCPAP pEECO_2_ was 2.4% and was higher than HFNC across all flows, but only achieved significance at 6–8 L/min (p<0.05).

### Effects of HFNC on tidal volume, ventilation and gas exchange

Reduction of HFNC from 8 to 2 L/min did not result in a change of weight-corrected tidal volume despite significant reduction in pEEP (table 2). Minute volume increased when flows reduced. Reducing flows from 8 to 2 L/min statistically significantly increased respiratory rate (p=0.047) and significantly lowered S_p_O_2_ by 4.4% (p<0.0001). Each 1 L/min flow rate increment improved S_p_O_2_ by 0.6%. Importantly, 13 subjects (30%) required FiO_2_ increased by 2%–9% when flows reduced from 8 to 2 L/min (eight were <1000 g, three were 1000–1500 g and two were >1500 g). TCCO_2_ was unchanged. Comparing nCPAP 6 cm H_2_O with HFNC 8 L/min at equal generated pEEP, HFNC 8 L/min resulted in similar weight-corrected tidal volume, TCCO_2_, SpO_2_ and heart rate (all p>0.05).

## Discussion

Key findings of our study were that flow rate was linearly related to pressure delivered, as suggested previously,^8–11 14 15 18^ and that weight, age, mouth position and prong-to-nares ratio are significant factors in determining pressure delivered. A substantial number of infants weighing <1000 g, in whom there is a paucity of previous data, were included. Furthermore, unlike previous studies,^8–12 14 15 19^ we included flow rates of 2–8 L/min that are commonly prescribed clinically.^1^ Previous data on pressures generated during HFNC are conflicting, likely due to different measurement techniques, small sample sizes and narrow flow rate protocols.^8–12 14 15 19^

Across all infants studied HFNC 8 L/min was comparable to 6 cm H_2_O nCPAP but average pEEP generated by HFNC of 6 L/min was lower than that generated by CPAP 6 cm H_2_O, which may be relevant to the recent finding in randomised studies that HFNC is inferior to nCPAP when used as primary support for preterm infants with respiratory distress syndrome.^4 5^ We also found considerable variability in pEEP generated at higher HFNC flows and at any given flow rate, the smallest infants received significantly higher pressures. Increased understanding of the mechanisms of action of HFNC in preterm infants should inform the design of future high-quality clinical studies.^20 21^

In our study, pEEP with the mouth closed was significantly higher than mouth open across all flow rates, similar to the findings of Arora *et al* in older infants with bronchiolitis.^14^ Previous neonatal studies have varied in results from no pressure generated when mouth open^9^ to no effect^8^ with work in an in vitro model^10^ showing that a leak as low as 30% leads to a dramatic reduction in pressure. Although not part of the study protocol, we observed that pEEP measurements were consistently lower when prongs were accidentally loosened highlighting the importance of correct positioning as per manufacturer’s instructions.

Generated pEEP correlated negatively with infants’ weight, a finding similar to some studies^8 9 18 22^ but not all.^13–15^ Importantly, 30 of our subjects were <1500 g, with 15 <1000 g. Some of the generated pEEPs (8–13 cm H_2_O) at higher flow rates were higher than those generated by 6 cm H_2_O nCPAP, contrasting with observations by Lavizzari *et al*,^19^ where only 75% of infants reached pEEP of 4 cm H_2_O and rarely >5 cm H_2_O. This may be due to our larger number of small infants and higher flow rates (>6 L/min). In infants weighing <1000 g, we found that flows as low as 4–6 L/min generate average pEEP similar to nCPAP 6 cm H_2_O and flows of 7–8 L/min delivered pEEP higher than nCPAP 6 cm H_2_O. Although rare, HFNC-related complications have been reported.^23 24^ Awareness of pressures delivered to vulnerable infants is important and may aid clinicians in prescribing flow rates. A recent survey found that 66% of clinicians adjusted flow in increments of 0.5–1 L/min when weaning: our data suggest that flow changes of 0.5 L/min are unlikely to have a major impact on respiratory parameters.^1^

Washout of nasopharyngeal dead space thereby increasing alveolar ventilation and improving CO_2_ elimination has been suggested as a mechanism of action of HFNC.^6^ This has been investigated in in vitro models,^25 26^ an animal study^16^ and adults^27 28^ but not in preterm infants. We found that increasing flows from 2 to 8 L/min led to significant reductions in pEECO_2_ and decreases in minute ventilation probably due to reductions in dead space ventilation, with the greatest effects seen in the smallest infants but without a significant change in TCCO_2_. Möller *et al* also demonstrated that dead space washout was flow-dependent,^26^ and reduction of CO_2_ rebreathing occurred during HFNC in tracheostomised adults.^27^ The pEECO_2_ was higher with nCPAP 6 cm H_2_O compared with all flow rates of HFNC supporting the hypothesis that HFNC reduces dead space better than nCPAP, similar to recent in vitro findings that washout times for nCPAP were significantly longer than HFNC by 16.2%.^25^ Our observation that mouth open was associated with lower pEECO_2_ measured compared with mouth closed was similar to previous work,^25^ suggesting that the shorter oral pathway surpasses the nasal route by providing the majority of the washout effect.

We acknowledge that the design of our study in vulnerable infants balanced patient safety as our overriding concern against acquisition of the best quality data possible in terms of invasiveness of measurements and timing at each level of respiratory support. There are significant limitations to the use of TCCO_2_ in premature infants,^29^ but it is non-invasive and arterial blood gas measurements would have been impractical. Our finding of a lack of change of TCCO_2_, which was within the normal range, during HFNC was similar to previous reports.^28 30 31^ We have also only investigated one HFNC and nCPAP delivery system.

The weight-corrected tidal volume measured across 2–8 L/min of flow and on nCPAP did not differ significantly, similar to previous reports.^19 30 32^ Explanations could be variability of sleep state in our infants as ventilatory responses to HFNC are different during wakefulness and sleep,^33^ and variability in infants’ need for non-invasive support at the time of study and age range. Increases in pEEP result in increases in functional residual capacity while tidal volume in infants may be more dependent on the degree of lung disease and work of breathing. Mauri *et al* recently demonstrated in adults that HFNC increases end-expiratory lung volume, but tidal volume was unchanged.^32^

We demonstrated that reducing flows from 8 to 2 L/min led to a significant increase in respiratory rate, in agreement with previous studies.^10 15 19^ Interestingly, we found that both respiratory and heart rate were generally higher during nCPAP therapy, possibly explained by better tolerance of HFNC. Increasing flows improved oxygenation saturation, as demonstrated previously.^18^

Although all infants tolerated the study protocol well, with no adverse events, 30% of participants (highest in the <1000 g group) required an oxygen increment to maintain their SaO_2_ within set parameters, which could have mitigated changes in some parameters but was essential to ensure safety. Without simultaneous oesophageal pressure measurement, we could not investigate compliance and work of breathing. However, adding this would have entailed significant additional handling, and an oesophageal pressure probe may have impacted on airway physiology and caused discomfort. Although the nasopharyngeal catheter used to measure pressure was similar to a nasogastric feeding tube, it is conceivable that it generated a degree of leak. However, HFNC apparatus are designed as ‘leaky systems’ to prevent barotrauma and the CPAP system used compensates automatically to maintain a set pressure.

In summary, multiple factors impact the pEEP delivered by HFNC in preterm infants, which leads to considerable variability. Extremely small infants are at greatest risk of receiving high pEEP. Physiological effects of increasing HFNC flow rate include raised airway pressure, improved oxygenation, lower respiratory rate and improved effective alveolar ventilation by reducing dead space ventilation.
