# Supplementary material for: Physiological effects of high-flow nasal cannula therapy in preterm infants
Source: Arch Dis Child Fetal Neonatal Ed. 2019 May 23;105(1):87–93. doi: 10.1136/archdischild-2018-316773 (PMC6951230; doi:10.1136/archdischild-2018-316773)
Supplement: Supplementary data [file fetalneonatal-2018-316773supp001.docx]

**DATA SUPPLEMENT**

**Physiological Effects of High-Flow Nasal Cannula Therapy in Preterm Infants**

Zheyi Liew^a,b,c^, Alan Fenton^b^, Sundeep Harigopal^b^, Saikiran Gopalakaje^a,b^, Malcolm Brodlie^a,c,*,#^, and Christopher O’Brien^a,*^

^a^Department of Paediatric Respiratory Medicine, Great North Children's Hospital, Newcastle upon Tyne Hospitals NHS Foundation Trust, Newcastle upon Tyne, United Kingdom;

^b^Newcastle Neonatal Service, Royal Victoria Infirmary, Newcastle upon Tyne Hospitals NHS Foundation Trust, Newcastle upon Tyne, United Kingdom;

^c^Institute of Cellular Medicine, Faculty of Medical Sciences, Newcastle University, Newcastle upon Tyne, United Kingdom.

*Equal contribution.

^#^Corresponding author: Malcolm Brodlie (email: [malcolm.brodlie@ncl.ac.uk](mailto:malcolm.brodlie@ncl.ac.uk), telephone: 0191 2821752), MRC Clinician Scientist/Honorary Consultant in Paediatric Respiratory Medicine, Level 3, Clinical Resource Building, Royal Victoria Infirmary, Newcastle upon Tyne, NE1 4LP. United Kingdom.

**List of elements in this data supplement**

1. Supplementary Methods
2. Supplementary Table S1 Characteristics of baseline respiratory support settings and oxygen requirement FiO_2_ at study entry
3. Supplementary Table S2 Relationship between nasopharyngeal end-expiratory CO2 and HFNC flow rate with mouth open and closed states
4. Supplementary Figure S1 Consort Diagram
5. Supplementary Figure S2 Schematic drawing of placement of nasopharyngeal catheter in the nasopharyngeal space
6. Supplementary Figure S3 Representative example of a measurement and data extraction of various physiological parameters and stable respiratory waveform in an infant receiving HFNC at 8L/min
7. Supplementary Figure S4 Example of airway pressure tracing with stable respiratory waveform for at least 10 breaths
8. Supplementary Figure S5 Prong-to-nares ratio (degree of nasal prong leak)

1. **Supplementary Methods**

**Study design**

A volunteer sample of stable infants <37 weeks gestation, aged >3 days and receiving nCPAP or HFNC for the preceding 12 hours were randomised to group 1 (nCPAP then HFNC) or group 2 (HFNC then nCPAP) using Qminim software (Sourceforge) to minimise bias.^1^ Infants were transitioned from their existing support of HFNC (2-8L/min) or nCPAP (4-6cm H_2_O) to nCPAP 6cm H_2_O (group 1) or HFNC 8L/min (group 2). Please see Figure 1 for further details. Inspired oxygen concentration (FiO_2_) was continued as before commencing the study.

## *Indications of clinical stability were*

1. Adequate ventilation from a recent blood gas: pH>7.2 and pCO_2_<10kPa
2. Stable oxygen requirement in the previous 12 hours – FiO_2_ increased less than 0.2 from baseline.
3. Stable observations including heart rate (100-180 per minute), respiratory rate (30-60 per minute) and temperature (36.5- 37.5^o^C)
4. No evidence of current infection: blood culture positive infection and or treatment for active infection.

## *Exclusion criteria*

1. Infants with known major congenital malformations or anomalies including of the upper airway, lower respiratory tract, cardiac or gastrointestinal tract
2. Infants who were clinically unstable and unsuitable for non-invasive respiratory support
3. Infants participating in a concurrent study that prohibited inclusion in other studies

## *Monitoring during the study*

Each infant was monitored continuously throughout the study. Any change in oxygen saturation (SpO_2_) outside the standard target range was addressed as per standard unit policy (FiO_2_ adjusted in 5% aliquots). A sustained increase (>15 minutes) in FiO_2_ by 20% above baseline or FiO_2_>60% triggered restoration of the infant to the previous mode of respiratory support. Significant apnoea was never observed.

## *Termination of study and significant apnoea definition*

1. Inadequate oxygenation – FiO_2_>0.6 and / or increase in FiO_2_ of 0.2 from baseline to maintain S_p_O_2_>91%.
2. Recurrent unprovoked minor apnoea requiring intervention (not self-corrected; >2 per hour during the study) or one major apnoea requiring positive pressure ventilation.

*Tidal volume measurements*

The VoluSense vest was applied over the torso >20 minutes before study commencement for adaptation. Subjects were studied supine.

**Equipment calibration**

*Pressure Transducer*

Pre-measurement two-point transducer calibration was performed using a water manometer. Positioning was confirmed by identification of a stable respiratory waveform. The frequency response of the differential pressure transducer (B&D Electromedical, range 0-30cm H_2_O) was tested with the nasopharyngeal catheter (Argyle Gentle Flow Suction Catheter, Size 8Fr, Covidien, Ireland) by the ‘pop’ test method.^2^ The response time was 20 milliseconds giving a frequency response of 16.7 hertz.

Gas Analyser: Calibration was performed using a known gas concentration source (12.5% O_2_, 7.5% CO_2_ mixed gas cylinder, British Oxygen Company).

Electromagnetic Inductance Plethysmography (VoluSense): Calibration was performed against a coil with known reference area positioned at the side of the infant to establish the relationship between the induced voltage and area, as per manufacturer’s instructions.

**Statistical methods**

SPSSv.23 (IBM) was used for statistical analysis. ANOVA or non-parametric equivalent Friedman, Wilcoxon signed rank, and Jonchkeere-Terpstra tests were used as appropriate based on normality, to compare means. Multiple comparisons after ANOVA were performed using the Tukey test. Spearman correlation test was performed. Multiple linear regression estimated the relationships between variables and predicted the value of generated nasopharyngeal end-expiratory pressure (pEEP) based on these variables.

**2. SUPPLEMENTARY TABLE S1**

**Characteristics of baseline respiratory support settings and oxygen requirement (FiO_2_) at study entry.**

|  | **Group 1** | **Group 2** |
| --- | --- | --- |
|  | Mean (range) | Mean (range) |
| Birth gestation (weeks) | 27.0 (23.6 – 31.6) | 27.0 (23.1 – 30.6) |
| Current gestation (weeks) | 32.9 (28.6 – 40.4) | 32.2 (28.3 – 42.1) |
| Age (days) | 44 (3 - 96) | 37 (4 - 132) |
| Birth weight (grams) | 880 (500 - 1900) | 900 (500 - 1620) |
| Current weight (grams) | 1470 (760 - 3000) | 1410 (610 - 4200) |
| Respiratory support at study entry | HFNC 2-6L/min | HFNC 2-6L/min |
| FiO_2_ at study entry (%) | 29 (21-45) | 28 (21-42) |
| FiO_2_ adjusted (number of subjects) | 7 | 6 |
| FiO_2_ increased (%) | 1 (2-9) | 2 (4-9) |
| Group 1 infants received nCPAP first then HFNC; Group 2 infants received HFNC first then nCPAP. 13 subjects (30%) required FiO2 increment between 2-9% during the study when HFNC 8L/min was reduced to 2L/min. 8 of these 13 subjects (62%) were preterm infants <100g, 3 infants were 1000-1500g and the remaining 2 infants were >1500g. | | |

**3. SUPPLEMENTARY TABLE S2**

**Relationship between pharyngeal end-expiratory CO_2_ and HFNC flow rate with mouth open and closed states**

| **HFNC flow rate (L/min)** | **Mean end-expiratory CO_2_** | | **Difference between open and closed** | **p Value*** |
| --- | --- | --- | --- | --- |
|  | **Mouth open** | **Mouth closed** |  |  |
| 2 | 2.14 | 3.16 | 1.02 | NS |
| 3 | 1.72 | 2.82 | 1.10 | NS |
| 4 | 1.52 | 2.10 | 0.58 | NS |
| 5 | 1.58 | 2.01 | 0.43 | NS |
| 6 | 1.41 | 1.42 | 0.01 | NS |
| 7 | 0.80 | 1.54 | 0.74 | NS |
| 8 | 0.81 | 1.12 | 0.31 | NS |
| *Wilcoxon signed rank test (NS = Not significant) | | | | |

**4. SUPPLEMENTARY FIGURE S1**

**Consort diagram**


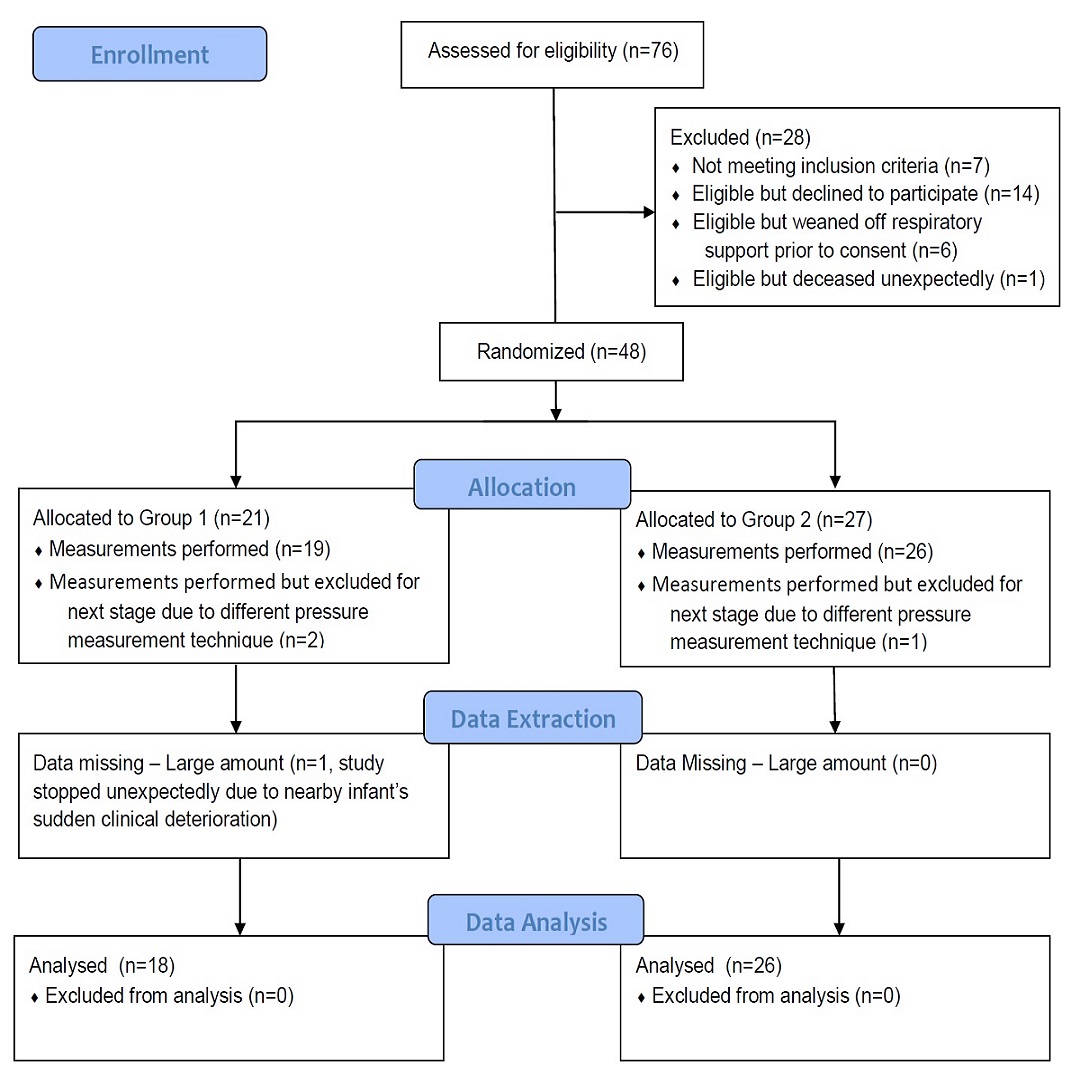


Preterm infants born below 37 weeks gestation, more than 3 days old on either HFNC or nCPAP respiratory support were screened for eligibility.

**5. SUPPLEMENTARY FIGURE S2**

**Schematic drawing of placement of nasopharyngeal catheter in the nasopharyngeal space**


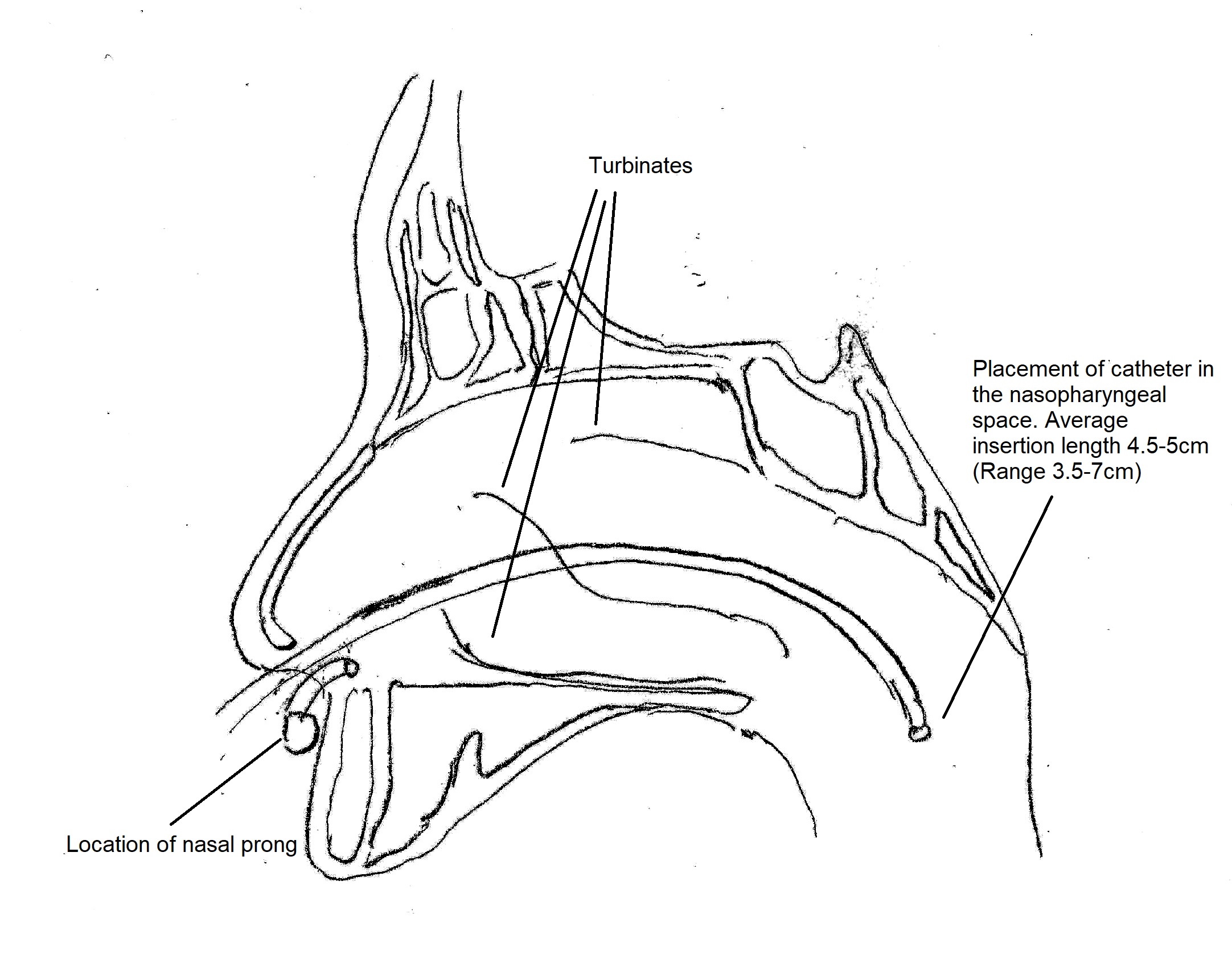


The placement of the catheter for each subject was consistent throughout the study. The distance between the nare of the nose to the tragus of the ear was measured before catheter placement. The insertion distance was 1cm less than this measurement to ensure adequate nasopharyngeal placement whilst avoiding stimulation of the gag or cough reflex. A 3-way tap allowed pressure measurement or gas analysis, the latter only performed with a stable respiratory pattern on pressure tracing.

**6. SUPPLEMENTARY FIGURE S3**

**Representative example of a measurement and data extraction of various physiological parameters and stable respiratory waveform in an infant receiving HFNC at 8L/min**


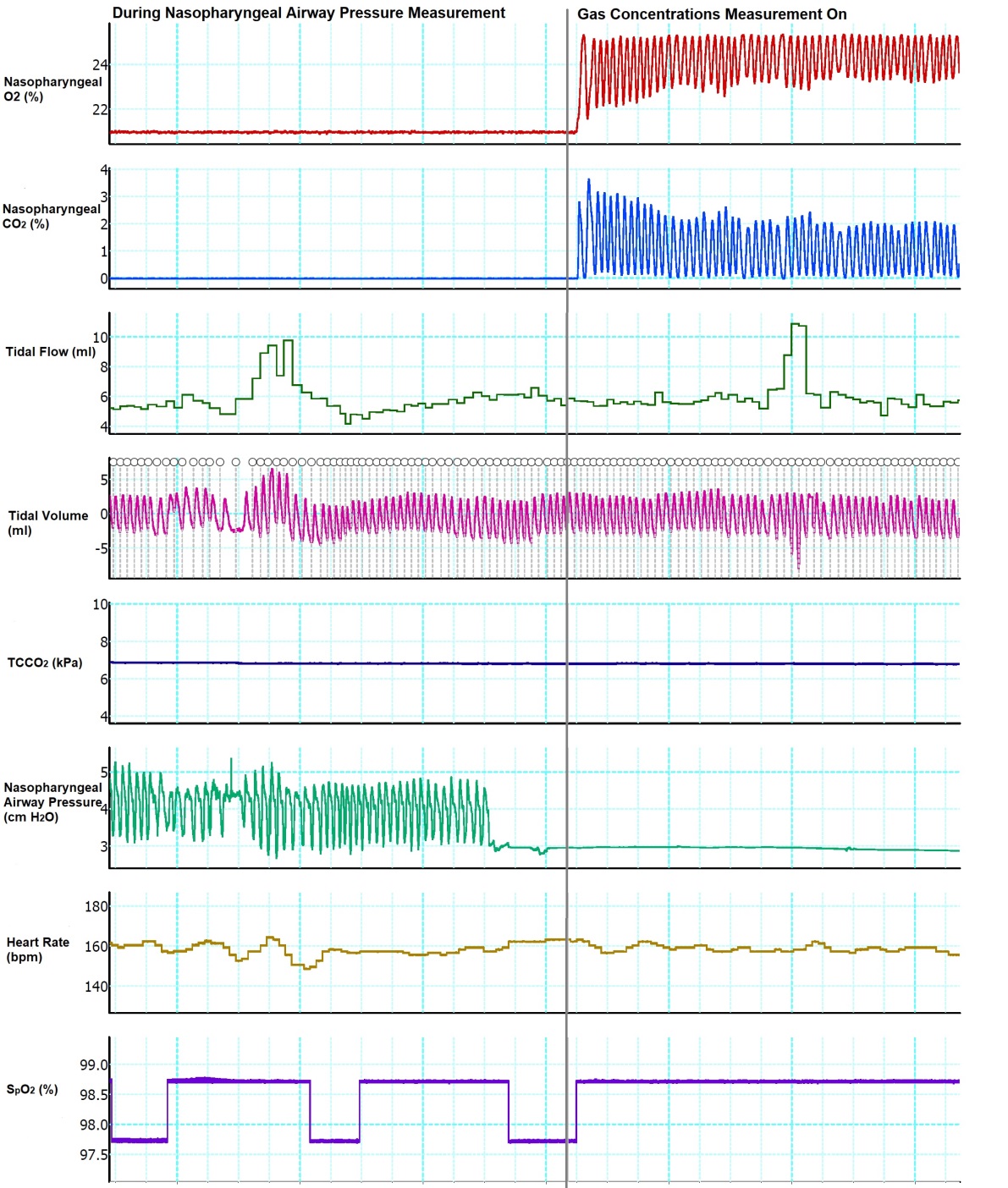


Respiratory rate was calculated from the continuous airway pressure waveform.

**7. SUPPLEMENTARY FIGURE S4**

**Example of airway pressure tracing with stable respiratory waveform for at least 10 breaths**


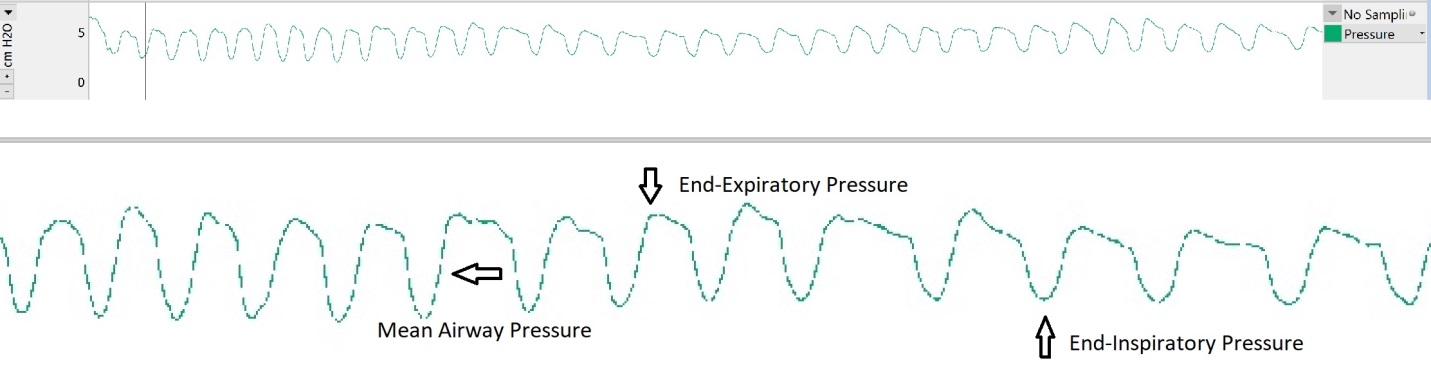


All artefact free data was extracted in blocks Each block extracted for analysis contained at least 10 consecutive breaths. On average, selected blocks contained 4 minutes 19 seconds of data extracted for each level of respiratory support, ranging from 14 seconds to 10 minutes 16 seconds for each block, and a median of 2 minutes 53 seconds per selected block.

**8. SUPPLEMENTARY FIGURE S5**

**Prong-to-nares ratio (degree of nasal prong leak)**


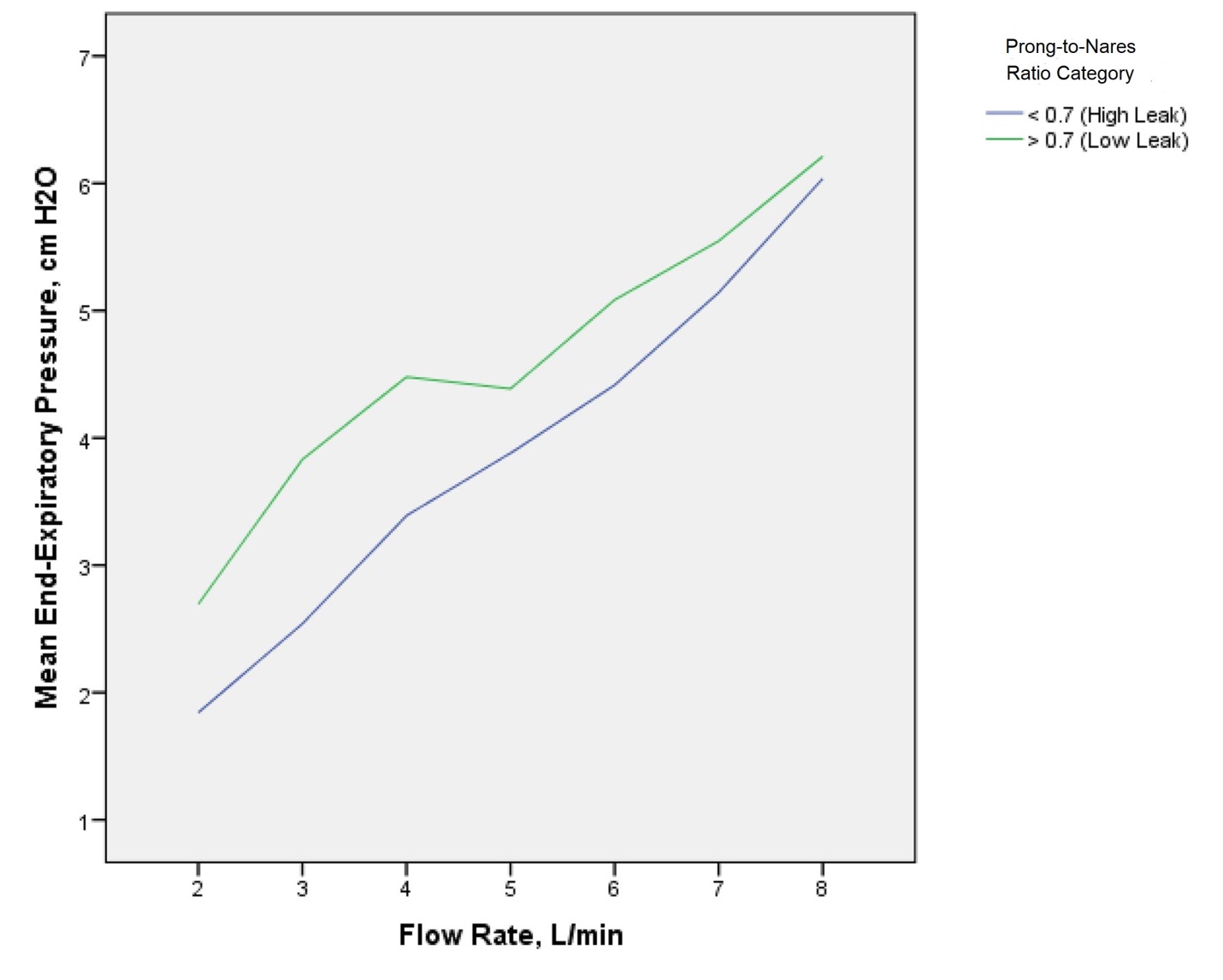


pEEP in the low-leak group compared to high-leak group (p<0.05 at flows 2-4L/min, Jonckheere-Terpstra test) Nasal prongs and diameter of nares were measured using standard measurement tape prior to the commencement of the study.

**REFERENCES**

1. Saghaei M, Saghaei S. Implementation of an open-source customizable minimization program for allocation of patients to parallel groups in clinical trials. *Journal of Biomedical Science and Engineering* 2011;Vol.04No.11:6. doi: 10.4236/jbise.2011.411090

2. Gabe IT. Chapter 2 - Pressure Measurement in Experimental Physiology. In: Bergel DH, ed. Cardiovascular Fluid Dynamics: Academic Press 1972:11-50.
